# Performance of Single Nucleotide Polymorphisms versus Haplotypes for Genome-Wide Association Analysis in Barley

**DOI:** 10.1371/journal.pone.0014079

**Published:** 2010-11-22

**Authors:** Aaron J. Lorenz, Martha T. Hamblin, Jean-Luc Jannink

**Affiliations:** 1 United States Department of Agriculture-Agricultural Research Service, R.W. Holley Center for Agriculture and Health, Cornell University, Ithaca, New York , United States of America; 2 Institute for Genomic Diversity, Cornell University, Ithaca, New York, United States of America; United States Department of Agriculture, Agricultural Research Service, United States of America

## Abstract

Genome-wide association studies (GWAS) may benefit from utilizing haplotype information for making marker-phenotype associations. Several rationales for grouping single nucleotide polymorphisms (SNPs) into haplotype blocks exist, but any advantage may depend on such factors as genetic architecture of traits, patterns of linkage disequilibrium in the study population, and marker density. The objective of this study was to explore the utility of haplotypes for GWAS in barley (*Hordeum vulgare*) to offer a first detailed look at this approach for identifying agronomically important genes in crops. To accomplish this, we used genotype and phenotype data from the Barley Coordinated Agricultural Project and constructed haplotypes using three different methods. Marker-trait associations were tested by the efficient mixed-model association algorithm (EMMA). When QTL were simulated using single SNPs dropped from the marker dataset, a simple sliding window performed as well or better than single SNPs or the more sophisticated methods of blocking SNPs into haplotypes. Moreover, the haplotype analyses performed better 1) when QTL were simulated as polymorphisms that arose subsequent to marker variants, and 2) in analysis of empirical heading date data. These results demonstrate that the information content of haplotypes is dependent on the particular mutational and recombinational history of the QTL and nearby markers. Analysis of the empirical data also confirmed our intuition that the distribution of QTL alleles in nature is often unlike the distribution of marker variants, and hence utilizing haplotype information could capture associations that would elude single SNPs. We recommend routine use of both single SNP and haplotype markers for GWAS to take advantage of the full information content of the genotype data.

## Introduction

Recent advances in sequencing and genotyping technology have allowed the collection of large amounts of genome-wide single nucleotide polymorphism (SNP) data for many species, primarily with the goal of finding associations between alleles and phenotypes of interest. Numerous statistical methods for such association studies have been proposed, many focused on the mapping of variation underlying common diseases in humans (e.g., Welcome Trust Case Control Consortium [1]), while others have focused on organisms as diverse as Arabidopsis [2], dogs [3] and cattle [4]. It has become apparent that the choice of association mapping methodologies depends on the characteristics of the study population. In contrast to biparental mapping populations, in which the pattern of LD and the allele frequency distribution are known and are independent of population genetic parameters, every association mapping population has a unique population history (both recent and ancient) that shapes its patterns of genetic variation and may determine which mapping method works best.

Patterns of variation can be described by the extent to which variation at linked SNPs is “block-like”, i.e., most haplotypes fall into a few classes with little evidence of recombination. This quality was first observed in dense SNP data from The SNP Consortium Allele Frequency Project [5], and has led to a great interest in determining whether the power and accuracy of association mapping can be improved by grouping SNPs into haplotype blocks (see Zhao et al. [6] for a review). Various rationales for testing for associations between phenotypes and haplotypes, rather than single SNPs, have been proposed, including that haplotypes: capture epistastic interactions between SNPs at a locus [7], [8]; provide more information to estimate whether two alleles are IBD [9]; reduce the number of tests and hence the type I error rate [2]; allow informed testing between clades of haplotype alleles by capturing information from evolutionary history [10]; provide more power than single SNPs when an allelic series exists at a locus [11].

These rationales may be more or less relevant depending on the marker density and LD structure of the data. For example, the possibility of epistasis among SNPs is much greater when SNP density is very high, and is particularly relevant to studies where many common SNPs have been typed across a candidate gene [7]. Using genotype and gene expression data from HapMap populations, Dimas et al. [12] have proposed that interactions between protein-coding and regulatory SNPs may be common, and there is some evidence for SNP-SNP interactions within genes and gene clusters (e.g., Hamon et al. [13]). But there is no evidence to suggest that epistasis occurs frequently between randomly chosen SNPs hundreds of kilobases apart.

Long and Langley [14], in a simulation study using parameters based on human data, concluded that “[o]ver the entire parameter space examined in this work and under the simple population genetic model considered, single-marker-based, permutation-based tests are either of similar or greater power than haplotype-based tests.” Since then, however, simulations based on the LD and population history of livestock have shown that haplotypes can provide greater QTL detection power and mapping accuracy than single markers can [15]–[17]. Zhao et al. [18], conducting simulations designed to resemble the demography and population history of livestock, found no apparent advantage to using haplotypes over single SNPs. These conflicting results suggest, as Long and Langley [14] noted, that “under different models relating genotype to phenotype or under different demographic scenarios, [their] conclusion may not be valid,” and that the power of QTL mapping with haplotypes must be evaluated on a case-by-case basis.

A GWAS report [19] as well as LD fine mapping studies [20], [21] using empirical data have found significant associations between haplotypes and phenotypes that were not detectable by a single SNP analysis. These results underscore a key difficulty in simulation studies: unless simulations accurately model the genetic architecture and population history of QTL alleles, they will have limited relevance to empirical datasets. Furthermore, it is likely that the nature of the QTL-marker associations is sufficiently variable that no one simulation approach can capture them.

Genome-wide association studies (GWAS) are now being used to identify genes underlying agronomically important traits in crops, many of which have self-pollinating mating systems. The objective of this study was to explore the utility of haplotypes for GWAS in barley, as a representative of such crops. To accomplish this, we used genotype and phenotype data from the Barley Coordinated Agricultural Project (Barley CAP; www.barleyCAP.org) for both simulations and analysis of empirical data. As for many plant studies, the current Barley CAP marker dataset is fairly sparse, with an average SNP spacing of about 2.4 Mb (2198 SNPs/5350 Mb genome), in a population of 1807 individuals. While this number of markers may seem too small to be of any value, linkage disequilibrium in this population is quite extensive in comparison to humans [22] or cattle [23], and there is substantial clustering of SNPs [24], so many adjacent SNPs are correlated. There are various criteria for defining haplotype blocks, with the most appropriate for a given case dependant on how the haplotypes will be used [6]. We used three different approaches to group sets of SNPs into haplotype blocks: the four gamete method as implemented in Haploview [25], based on recombination; the HapBlock method [26], based on diversity; and a simple sliding window. For each one of these approaches, we tested the power to detect associations between haplotype alleles and simulated QTL. In addition, we used the blocks defined in the four gamete method to construct parsimony trees, and tested the power to detect associations between the simulated QTL and edges in the trees, as proposed by Templeton et al. [10] and implemented in the TreeScan software [27]. Finally, association analysis using the haplotypes, parsimony tree edges, and single SNPs was conducted on heading date data collected on a large set of barley germplasm from the Barley CAP.

## Results

### SNP data

The mean genetic map distance between adjacent markers was ∼0.5 cM, though 60% of adjacent SNP pairs had the same map position. When markers mapped to the same position, it was most likely because of insufficient resolution of bi-parental maps rather than because of actual identical positions [28]. The distributions of minor allele frequency (MAF), mean *r^2^* between adjacent SNPs, and the highest *r^2^* within 10 cM, are shown in Table 1. Because the complete sequence of the barley genome is not available, the physical distance between markers is not known.

**Table 1:** Properties of SNPs scored for the Barley Coordinated Agricultural Project with minor allele frequency ≥0.028.

	Min	Median	Mean	Max
Minor allele frequency	0.028	0.308	0.290	0.50
*r^2^* between adjacent SNPs	0	0.378	0.453	1.00
Max *r^2^* within 20 cM window	0.009	0.684	0.640	1.00

### Haplotype Blocks

We identified haplotype blocks using three methods based on different properties of the data. Properties of the blocks are shown in Table 2. Both the 4gamete and HapBlock methods produce blocks that vary greatly in size, depending on regional properties of the data (i.e., linkage disequilibrium and marker density). About 26% of the block boundaries are shared between the 4gamete and HapBlock methods; across the genome, 38 of the blocks are identical. To contrast with these methods, we also grouped SNPs by a simple sliding window approach. Because the median block size of the other two methods was three SNPs, we used a block size of three SNPs (SlideWin3).

**Table 2:** Comparison of single SNPs and haplotype blocks inferred by the three blocking methods.

	BOPA1&2^a^	4Gamete	HapBlock	SlideWin3
Number of loci^b^	2098	791	585	2084
Single SNPs remaining	2098	323	37	0
Total alleles	4196	2584	2762	8320
Alleles (MAF^c^≥.028)	4196	2301	2484	6002
Effective test number	1164	744	521	791
Mean *H_e_* d	0.372	0.501	0.593	0.584
Mean major allele freq.	0.71	0.54	0.52	0.54
Proportion of genetic map in blocks	na	47%	63%	100%
**Block size in cM**				
Mean	na	1.09	1.25	1.04
Median	na	0.72	0.76	0.70
Variance	na	1.97	2.14	1.83
Maximum	na	10.19	11.06	10.35
**Block size in SNPs**				
Mean	na	3.80	3.82	3
Median	na	3	3	3
Variance	na	6.28	5.99	0
Maximum	na	20	30	3

a SNPs scored using two Barley oligonucleotide pool assays (Close et al. 2009).

b Includes unblocked SNPs.

c MAF, minor allele frequency.

d *H_e_*, expected heterozygosity.

The 4gamete method resulted in the fewest SNPs being included in blocks (85%), and less than half of the genetic map was covered by blocks. In contrast, the SlideWin3 method covered the entire genetic map, because the blocks were overlapping. The HapBlock method produced the largest average number of alleles per locus (4.7), consistent with its higher average block size in cM. The lower average alleles per locus for the 4gamete method was mainly due to the large number of singleton (i.e., unblocked) SNPs.

### Power to detect single SNP-based QTL

**Figure 1:**
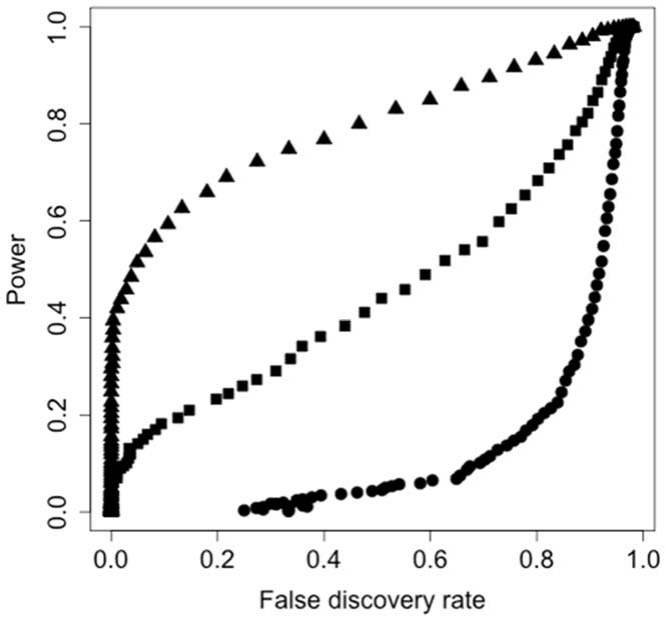
Power plotted against false discovery rate for the single SNP analysis and each level of QTL size (*p*). Triangles represent *p* = 0.12, squares represent *p* = 0.06, and circles represent *p* = 0.03. For all QTL sizes *h^2^* = 0.75.

Power to detect single causal SNPs (QTL) removed from the marker dataset for each QTL size (*p*) and *h^2^* is presented in Table 3. Substantial power to detect QTL at reasonable false discovery rates (FDRs) was observed only when *p* was set to 0.12 (Table 3; Figure 1). At an FDR of 0.1, power to detect *p* = 0.03 QTL was 0.01 or less in all cases, while power to detect *p* = 0.06 QTL was 0.09 and 0.18 at *h^2^* levels 0.25 and 0.75, respectively. Increasing the proportion of phenotypic variation caused by the polygenic effect (i.e., *h^2^*) increased power at constant QTL size because the K matrix, which describes the genome-wide genetic covariance between individuals, can account for more of the phenotypic variation. This produces a clearer QTL signal. The relationship between power and FDR for each QTL size is presented in Figure 1.

**Table 3:** Power of detecting single QTL removed from marker dataset for different heritability and QTL size levels and empirical false-discovery rates.

False-discovery rate = 0.10					
	Single SNP	4gamete	SlideWin3	HapBlock	TreeScan
*h^2^*, *p*					
0.25, 0.03	0.00	0.00	0.00	0.00	0.00
0.25, 0.06	0.09	0.05^a^	0.08^a^	0.04	0.07^a^
0.25, 0.12	0.43	0.44	0.57	0.46	0.43
0.75, 0.03	0.00	0.00	0.00	0.00	0.00
0.75, 0.06	0.18	0.10	0.17^a^	0.11	0.11^a^
0.75, 0.12	0.57	0.54	0.73	0.57	0.49

False-discovery rate = 0.20					
	Single SNP	4gamete	SlideWin3	HapBlock	TreeScan
*h^2^*, *p*					
0.25, 0.03	0.00	0.00	0.00	0.00	0.00
0.25, 0.06	0.11	0.08^a^	0.11	0.07^a^	0.08^a^
0.25, 0.12	0.53	0.56	0.68	0.60	0.53
0.75, 0.03	0.00^a^	0.00^a^	0.01	0.00^a^	0.00^a^
0.75, 0.06	0.24^a^	0.16	0.27	0.16	0.17
0.75, 0.12	0.68	0.63	0.82	0.67	0.58

a Powers not significantly different than highest power within row at 0.01 probability level.

Numerically greatest values within a row are underlined.

Power was largely equivalent between the different analysis methods when *p* = 0.06 and 0.03 and *h^2^* = 0.25. When *h^2^* = 0.75 and *p* = 0.06, the single SNP and SlideWin3 analyses provided more power at both FDRs. SlideWin3 displayed a substantial advantage over all other methods when *p* = 0.12. The advantage ranged from 20% to 33% over the next best method.

### Power to detect haplotype-based QTL

Simulating QTL by assigning a phenotypic value to a genotyped SNP marker assumes that the frequency distribution of QTL is similar to that of markers. To simulate a scenario in which the causal variant is younger than the marker variants, pairs of SNPs at the center of haplotype blocks were chosen and a phenotypic effect was assigned to lines carrying a specific combination of alleles at those loci (see *Methods*). Because we found that *p* = 0.06 and *h^2^* = 0.75 best separated the different analyses when QTL were simulated as removed, single causal SNPs, these parameter levels were used for this second round of simulations. With few exceptions, each analysis provided the highest power only when the corresponding block structure was used for simulating the QTL (Table 4). For example, when QTL simulations were based on pairs of SNPs at the center of HapBlock haplotype blocks, QTL were detected most effectively by performing the association analysis with HapBlock haplotypes. An important observation to note is that SlideWin3 almost always resulted in the second best power, and sometimes even numerically better than the haplotype analysis method matching the QTL simulation method, e.g. 4gamete QTL at FDR = 0.20 in Table 4.

**Table 4:** Power of detecting single causal SNPs left in the marker dataset or QTL pairs chosen on the basis of the different block methods (rows) for each analysis method (columns).

False-discovery rate = 0.10					
	Single SNP	4gamete	SlideWin3	HapBlock	TreeScan
QTL type					
Single SNP	0.51	0.34	0.39	0.29	0.41
4gamete	0.23^a^	0.30	0.24^a^	0.18	0.21
SlideWin3	0.10	0.12	0.27	0.14	0.08
HapBlock	0.19	0.14	0.23^a^	0.31	0.14

False-discovery rate = 0.20					
	Single SNP	4gamete	SlideWin3	HapBlock	TreeScan
QTL type					
Single SNP	0.59	0.41	0.48	0.37	0.48
4gamete	0.31	0.42^a^	0.44	0.29	0.28
SlideWin3	0.15	0.20	0.44	0.24	0.11
HapBlock	0.25	0.23	0.41	0.41	0.19

a Powers not significantly different than highest power within row at 0.01 probability level.

Numerically greatest values within a row are underlined.

### Heading date association mapping

**Figure 2:**
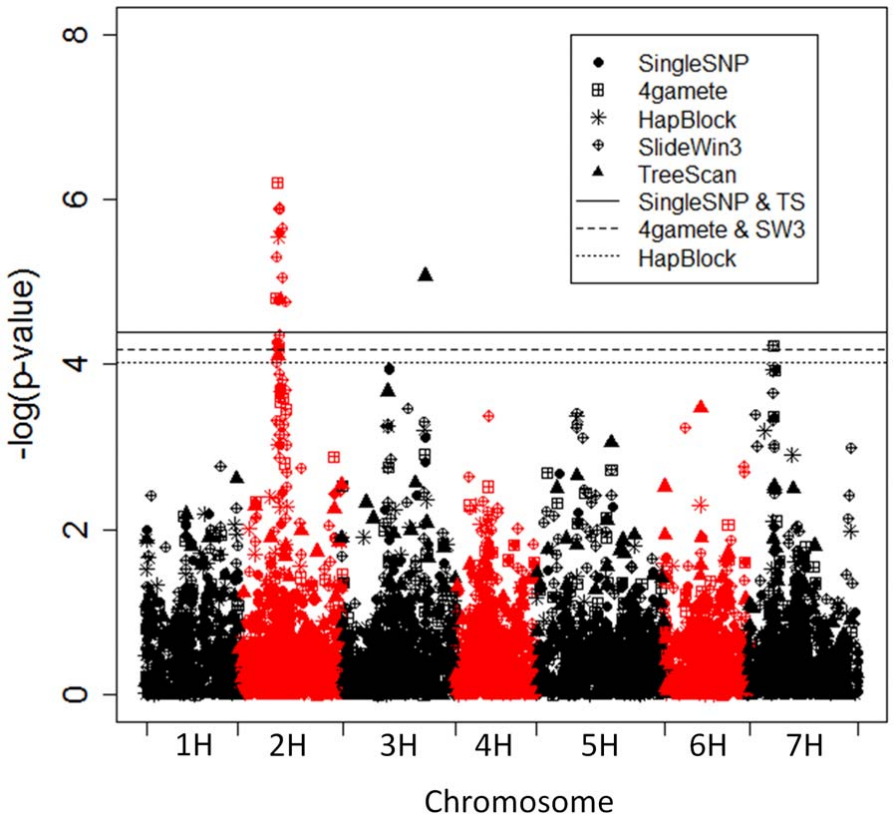
Manhattan plot showing significance of each marker. Markers are plotted on the x-axis according to their genetic position on each chromosome. Horizontal solid, dashed, and dotted lines correspond to an experiment-wise error rate of 0.05 for the single SNP, 4gamete, SlideWin3 (SW3), HapBlock, and TreeScan (TS) analyses as indicated in the legend.

We conducted genome-wide association mapping on heading date in the Barley CAP germplasm using the same single SNP and haplotype analyses performed on the simulated phenotypes. Across all analyses, three associations were found between markers and heading date (Figure 2), two of which were detected by haplotype analyses only. The strongest association was for markers on the long arm of chromosome 2H near the centromere (position 63.53 cM on the consensus map of Close et al. [28]). This region has been referred to as Qrgz-2H-8 [29] and is typically associated with QTL for heading date and Fusarium Head Blight resistance [30]. Microsatellite marker *GBM1023*, used by Nduulu et al. [30] for genetically dissecting this region, is linked to the SNP highly associated with heading date in this study, POPA2_1399 [28].

**Figure 3:**
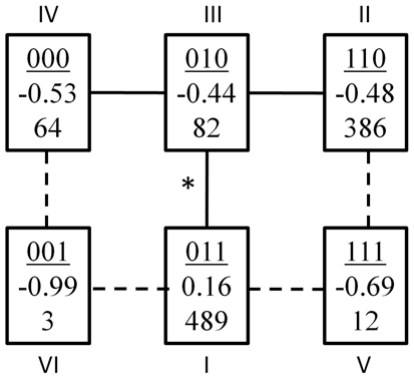
Parsimony tree of alleles present at 4gamete block 325 (chromosome 3, position 126.3). Values in boxes represent, from top to bottom, SNP scores each allele, best linear unbiased estimate of allelic effect, and number of lines carrying that allele. The edge separating alleles 010 (III) and 011 (I) was associated with heading date in the TreeScan analysis. Alleles VI and V were recombinant alleles not included in the parsimony tree and therefore the dotted edges were not included in the TreeScan analysis.

Working across Figure 2, the next marker-heading date association occurred on chromosome 3H, position 126.3. Interestingly, this association was found by the TreeScan analysis only. Figure 3 shows the configuration of the parsimony tree constructed with the alleles of this 4gamete haplotype block. There was a significant difference between allele 011 and the average of alleles 000, 010, and 110. The SNP at position 3 in this block separates 011 from the other alleles in this tree, but this SNP also groups 011 with 001 and 111, which are recombinant alleles that do not fit in the parsimony tree displayed in Figure 3. The allelic effects of these two recombinant alleles are quite different than 011. Despite a small number of individuals carrying the recombinant alleles, they influenced the effect of the SNP at position three by enough to increase the p-value to above the significance threshold. The p-value for the SNP at position 3 was 3.7×10^−4^, while that of the TreeScan edge was 8.5×10−6. The p-value for the 4gamete analysis was 5.5×10^−4^, illustrating that grouping lines with alleles 000, 010, and 110 together and performing a single degree of freedom test provided more power than the 4gamete multi-degree of freedom test. The SNP at position three in this block (POPA2_0650) maps very near to markers found to be associated with heading date in barley in at least three prior mapping studies. These include RFLP *ABG377*, reported to be associated with heading date by Hayes et al. [31] and Thomas et al. [32], and microsatellite *Hvm33* by Pillen et al. [33].

An association was found on chromosome 7H solely by the 4gamete and SlideWin3 analyses. The three alleles at this locus and best linear unbiased estimates of their allelic effects (in parentheses) were: 101 (0.735), 010 (0.728), and 011 (0). Grouping lines by any of the individuals SNPs in this haplotype block groups lines with different phenotypes, nearly eliminating any power to detect this association. Accordingly, p-values for the single SNP analysis in this region were much higher than the haplotype analyses (Figure 2). The number of individuals carrying allele 011 was 145. These lines were distributed across four breeding programs as follows: Aberdeen, 30 lines; NDSU 2-row, 73 lines; Washington State, 42 lines. The three SNPs that composed this haplotype block (POPA3_0893, POPA3_0894, POPA3_0895) were included on the Barley CAP genotyping platform because they are located within *VRN-H3*, a cloned gene controlling vernalization response that is orthologous to Arabidopsis Flowering Locus T [34]. Yan et al. [34] showed that variation exists in the promoter region of this gene independent of growth habit and could not rule out the possibility that this locus contributes to variation in flowering time within barley growth habit.

As mentioned in the introduction, one advantage of grouping SNPs into haplotype blocks is reducing the number of tests and making it easier to reject the null hypothesis. Because tests involving markers within linkage groups are not independent of one another, and the distribution of markers across the genetic map is not uniform, the effective number of independent tests is not necessarily linearly related to the number of markers (i.e., the number of loci as reported in Table 2). Using permutations, we determined the significance threshold required for an experiment-wise error rate (EER) of 0.05. The p-value thresholds were related to marker number, with the single SNP and TreeScan thresholds being the most stringent and the HapBlock threshold, with the fewest markers (Table 2), being the least stringent (Figure 2). The Bonferroni correction for multiple tests calculates the p-value that should be used for individual tests in order to maintain a desired EER assuming independent tests. Since our tests are not independent, it would be useful to calculate the effective number of independent tests to illuminate the degree to which grouping linked SNPs into haplotypes reduces the problem of multiple testing. We solved the Bonferroni function for test number and found the following effective independent test number for the various analyses: single SNP, 1164 tests; 4gamete, 744 tests; SlideWin3, 791 tests; HapBlock, 521 tests; TreeScan, 1306 tests. The concept discussed above is illustrated by the difference in effective test number between SlideWin3 and single SNP. These two methods have nearly the same number of loci because each SNP, with the exception of those on chromosome ends, gives rise to a new haplotype block in SlideWin3. Adjacent SlideWin3 blocks, however, share two SNPs and are therefore correlated more strongly with one another compared to adjacent single SNPs. This shows that although overlapping sliding windows generate nearly as many loci as single SNPs, it is still useful for reducing the problem of multiple testing.

## Discussion

The dramatically increasing availability of DNA markers will produce a landslide of genome-wide association mapping studies in crop species in the coming years. Our goal was to identify which analytical methods perform best in discovering genes controlling complex traits in crop germplasm collections so that allelic diversity can be mined most efficiently. Studies in other organisms have shown that, under certain conditions, multimarker SNP haplotypes may provide increased power to detect QTL. However, those conditions are not well understood, and the population genetic characteristics of plant species may differ in important ways from human and animal species, e.g., many plants are self-pollinating.

### Properties of blocks

The first issue we faced was the choice of blocking methods, of which a large number have been proposed in the literature. The vast majority of those methods were designed for application to human data sets with high densities of markers; the density of SNP markers in the BarleyCAP data set is much lower. However, in spite of this low density, there is significant LD between adjacent markers, due both to the substantial clustering of SNPs on the genetic map and to LD that extends over at least 10 cM [24]. We explored the use of two methods, based on LD (4gamete) or diversity (HapBlock), to assign SNPs to blocks; using the settings we implemented, 85% or 98% of SNPs were incorporated into blocks, respectively. Although these two methods grouped a large fraction of SNPs in blocks, when the genetic map distance between blocks was tallied, 37% and 53% of the genetic map fell between block boundaries (Table 2). A similar proportion of our simulated QTL also fell between block boundaries: 63% of the QTL fell between 4gamete blocks and fell 39% between HapBlock blocks. On the other hand, the sliding window method grouped all SNPs into blocks. Because the sliding windows were overlapping, there was no map distance left between blocks as well as no simulated QTL between blocks.

### Simulations with single SNPs as QTL

To compare the power of haplotypes and single SNPs in finding QTL, we simulated QTL at known genetic locations using 100 evenly distributed SNPs removed from the marker data set. We found that haplotypes did not provide an advantage at smaller QTL sizes, but at the largest QTL size investigated, SlideWin3 provided substantially more power than the single SNP analysis (Table 3). TreeScan analysis performed significantly worse than the single SNP analysis when heritability and QTL effect size were high. We considered several technical factors that could contribute to the small differences in power observed in some situations:

1. Window size for declaring a true positive (favors haplotypes). For the single SNP analysis, we required a significant marker to be within 10 cM of the causal SNP in order for it to be declared a true positive. For the haplotype analyses, we required a block boundary to be within 10 cM of the causal SNP; since the blocks covered a genetic distance up to 11 cM (Table 2), a haplotype could be farther away from the QTL and still be declared a true positive.
2. Test number (favors haplotypes). By grouping SNPs into haplotype blocks, the number of tests was reduced, reducing the probability of spurious associations. According to our permutations of the heading date data, an EER of 0.05 can be maintained at a slightly lower significance threshold for the 4gamete, SlideWin3, and HapBlock analyses. The less stringent significance threshold should provide greater power.
3. Test degrees of freedom (favors single SNPs). Because haplotypes are multi-allelic, the likelihood ratios calculated for them follow chi-squared distributions with more than 1 degree of freedom. For the same p-value, haplotype likelihood ratios must therefore be higher than single SNP likelihood ratios.
4. Allele frequency spectrum (favors single SNPs). All the 2098 single SNPs had MAF≥0.028. When these SNP loci were combined into haplotypes, they generated alleles that were of lower frequency. This was especially true of the SlideWin3 method, for which ∼28% of the alleles had MAF below 0.028. Elimination of these low MAF alleles resulted in smaller sample size and loss of information. Because of the generation of lower frequency alleles, larger sample sizes are needed to take advantage of the additional information present in haplotypes.
5. Amplification of missing data (favors single SNPs). When any SNP allele in a block is missing, the allelic state of the block is unknown for that individual, so there is inevitably more missing data in a haplotype data set than there was in the original SNP data set. In our case, we imputed the 0.7% of missing data for the SNPs, which resulted in 1.65%, 2.8%, 3.4%, and 2% imputed haplotypes for the 4gamete, TreeScan, HapBlock, and SlideWin3 methods, respectively.
6. Possible map order errors. Because the barley genetic map has clusters of markers with the same genetic map position, the order of some markers is unknown. As described in Methods, we used an approach that maximizes LD between adjacent markers to order these clusters. Errors in map order would have no effect on the single SNP analysis, but may lead to inference of haplotype alleles that do not actually exist. It is not clear what effect this would have on QTL detection power.

The differences in power that we observed between the methods are the net, combined effect of these technical factors as well as any differences in information content (i.e., marker-QTL LD) of the various marker types. As evaluated by the power/FDR relationships summarized in Table 3, this net effect is apparently close to zero for some of the methods and situations, but comes out in favor of SlideWin3 for large QTL size. To better understand the relative importance of technical factors versus information content for detection power, we compared the information content of the various marker types. We calculated *r^2^* (or its multiallelic equivalent, χ^2^′ [35]) between each of the 100 QTL and the markers within 10 cM, for each method. This maximal *r^2^* should strongly affect detection power of a method. Comparisons among the methods are shown in Table 5. While the LD between single SNPs and QTL is highly correlated with the LD between haplotype blocks and QTL (the correlations range from 0.84 to 0.90), for 63 of the QTL, at least one haplotype method generates a block with χ^2^′ at least 0.1 units higher than *r^2^* between that QTL and the best single SNP. At 15 QTL, all three haplotype methods produce markers with χ^2^′ at least 0.1 units higher than *r^2^* for any single SNP. For only 13 QTL is the *r* ^2^ for the best single SNP 0.1 higher than the maximum χ^2^′ of any of the blocking methods. In no case was a QTL more strongly associated to a single SNP than to a block from more than one haplotype method. That haplotype alleles were in higher LD with QTL than were the single SNP but often did not provide greater detection power indicated that, overall, the technical factors worked against the haplotype methods.

**Table 5:** Linkage disequilibrium between QTL and its highest LD marker for single SNPs and each of the different blocking methods.

	Single SNP	4Gamete	HapBlock	SlideWin3	TreeScan	Mean LD	Std. Dev. of LD
Single SNP	-	13	0	0	22	0.50	0.24
4Gamete	20	-	3	1	36	0.53	0.23
HapBlock	49	47	-	8	60	0.62	0.21
SlideWin3	55	47	6	-	65	0.63	0.21
TreeScan	1	9	0	0	-	0.43	0.27

Each cell shows the number of times the method in the row produced a marker with marker-QTL LD (i.e., χ^2^′ or *r^2^*) at least 0.1 units higher than the marker-QTL LD produced by the method in the column. Mean and standard deviation of LD are given in the last two columns.

Examination of Table 5 also provided a hypothesis for the greater increase in power of the SlideWin3 method for QTL of large effect. First we note that, with the exception of the TreeScan method, all haplotype methods increased in power more than the single SNPs as QTL size went from 0.06 to 0.12. Likewise, the variance across all QTL in their maximal LD with a marker was lower for haplotype methods (with the exception of TreeScan) than for the single SNPs (Table 5). For QTL of size 0.06, the median likelihood ratio was below the detection threshold (i.e., power was below 50%). In that case, having a higher variance in LD was beneficial because it caused a higher variance in the likelihood ratio such that in more cases the ratio exceeded the threshold. In contrast, for QTL of size 0.12, the median likelihood ratio was above the threshold. Higher variance was then detrimental because it caused more cases to fall below the threshold.

### Haplotype-based QTL and empirical phenotype data

Our analyses using phenotypes simulated on the basis of single SNP genotypes suggested that there can be an advantage to using haplotypes instead of single SNP markers in GWAS. Moreover, it is likely that the method of simulating the QTL biased the results in favor of the single SNP analysis. There is no reason to assume that the QTL alleles found in nature – the only relevant QTL – are distributed in populations and across the genome the same as SNPs placed on an Illumina GoldenGate SNP chip. To determine the effect of QTL simulation on our results, we performed three additional sets of simulations in which no SNPs were removed, and QTLs were assigned to a pair of adjacent SNP alleles (see *Methods*). This approach confirmed that, when the causal SNP is one of the genotyped markers, the power of single SNP analysis is superior to that of haplotypes (Table 4). However, when the QTL effect was simulated by a combination of SNPs, the blocking method that combined that pair had the greatest power to detect it in most cases. A QTL such as this may occur in nature when the age of causal polymorphism is younger than the surrounding marker polymorphisms [36]. If the haplotype containing the causal mutation subsequently increases in frequency through drift or selection, the QTL allele would be in higher LD with the surrounding haplotype as a whole than with any of the single SNPs within the haplotype. In this scenario, the increased information content of the haplotype alleles clearly offset any loss of power associated with technical issues.

The results of the heading date analysis are consistent with those from the QTL simulations: different marker-trait associations were found across different analyses, reflecting the heterogeneous history of mutation, recombination, and drift across the barley genome. The power to detect a QTL is highest when the associated marker allele has a similar frequency to that of the QTL allele; when a QTL allele is in lower frequency than the nearby markers, a combination of alleles at two or more markers may generate a haplotype that is closer in frequency. This appears to be the case for the QTL on chromosome 7H, which was in higher in LD with a 4gamete haplotype at *VRN-H3* than with any single SNP within this block. In an equilibrium population, this situation is most likely when the mutation giving rise to the QTL is younger than the mutations giving rise to the markers. In a domesticated species that has experienced at least one bottleneck and strong selection, allele frequency is not necessarily a reflection of allele age, and such a configuration can also be due to genetic drift.

The basis of the association between heading date and a TreeScan edge on chromosome 3H is somewhat more complicated. The increased power of the TreeScan method at this locus was due to 1) the higher power of a test with one degree of freedom and 2) the elimination of the phenotypically discordant recombinant alleles from the set of individuals that carry allele 1 at the most strongly associated SNP (Figure 3). Had we included these recombinant alleles in the parsimony tree, this would not have changed the result, namely, the significance of the edge between haplotype I and haplotypes II, III, and IV. However, the results in Table 5 suggest that this type of scenario favoring TreeScan does not happen frequently. Given the considerable effort involved in implementing the TreeScan method, which was originally developed for candidate gene analysis and cannot easily be automated, we do not recommend its routine use in GWAS.

It is worth mentioning that all the QTL detected for heading date were found by at least one of the haplotype analyses; even the peak on chromosome 2 (Figure 2) was topped off by an association with a 4gamete haplotype. However, the reasons for potential greater power of the single SNP analysis listed above, combined with the fact that no extra effort is required for performing the single SNP analysis, warrant its continued importance in GWAS.

### Conclusions

Because both genetic architecture and population history are likely to differ across genes and traits, it is not reasonable to expect one method to be superior at detection of all QTL. The examination of LD around individual simulated QTL shows that, in some cases, multi-SNP haplotypes can be in much stronger LD with a QTL-SNP than are any of the constituent SNPs. Both our simulation and barley heading date results provide good support for the use of simple, overlapping sliding windows for GWAS to complement a single SNP analysis. Fortunately, the sliding window haplotype method is also the easiest to implement, as it is implemented in the available software programs PLINK [37] and TASSEL [38]. Whether haplotypes would provide any advantage when marker density is much higher remains to be determined.

## Materials and Methods

### Genotyping

SNP data consisted of 3072 SNPs scored on 1824 barley lines using two Illumina GoldenGate oligonucleotide pool assays (BOPA1 and BOPA2 in Close et al. [28]). Barley lines were from CAP years 2006 and 2007 only. Unmapped SNPs and those with MAFs<0.028 were removed from the data set (a minimum of 50 individuals carried the minor allele). Since barley is highly inbred, the genotypic data were treated as effectively haploid. Heterozygous loci were rare and were scored as missing data. After removing duplicate lines and lines with large amounts of missing data, the final data set consisted of 2198 mapped SNPs scored in1807 lines.

There were many sets of SNPs, ranging in size from two to 34 SNPs, with identical genetic map position; these sets were ordered so as to generate maximal LD among adjacent markers. For example, if markers a, b, c, d, e, f were at 8, 10, 12, 12, 12, 15 cM, the best order would be chosen from a-b–[c, d, or e]- [c, d, or e]- [c, d, or e]-f by maximizing LD between b - [c, d, or e] and f - [c, d, or e] as well as LD among c, d, and e.

### Haplotype Block Identification

Three methods were used to identify haplotype blocks among the SNPs:

1. The four gamete method (4gamete), implemented in the software Haploview [25], creates block boundaries where there is evidence of recombination between adjacent SNPs, based on the presence of all four gametic types. We used a cutoff of 2%, meaning that if addition of a SNP to a block resulted in recombinant alleles at a frequency exceeding 2%, the SNP was not included in the block.
2. The HapBlock method [26] groups SNPs into blocks on the basis of diversity rather than LD: SNPs are grouped so as to capture most of the diversity across the sample in a set of common haplotypes, where “common” is defined by the user. We chose to identify blocks for which at least 97% of individuals have a haplotype allele that is present at a frequency of at least 2.8%.
3. Overlapping sliding windows of three SNPs were blocked together (SlideWin3).

Haplotype block identification was conducted on the original genotype data, which included 0.7% missing data. Because some of our analysis methods would not tolerate missing data, we used FastPHASE [39] to impute all missing SNP alleles. This program uses haplotype clustering that changes with genetic map position and allele frequencies to calculate a probability that an individual carries the reference allele at a locus. For each blocking method, we extracted the SNP boundaries and common alleles (frequency ≥0.02) identified using the original data and applied this information to the dataset with the imputed data, creating a haplotype allele incidence matrix that contained the probabilities each individual carried allele i at haplotype block j. The haplotype allele incidence matrix used for the association analysis was created as follows:

*k* columns were created for each haplotype block, where *k* is the number of alleles with frequency ≥0.02, and allele 1 is the most frequent. In addition, each unblocked SNP was represented by one column, as in the single SNP analysis. For each block, columns 1 to (*k*−1) store the probabilities that an individual carries alleles 2 through *k*, respectively. Column *k* stores the probability that an individual carries a minor allele. In the case that an individual carries allele 1, the row values for that block will be all 0s. If an individual is missing data for at least one SNP within a haplotype block, at least one column among the set of columns representing that block will have a value between 0 and 1, since the probabilities of various haplotype alleles are the products of the probabilities of the component SNPs as determined by the imputation procedure.

### TreeScan

**Figure 4:**
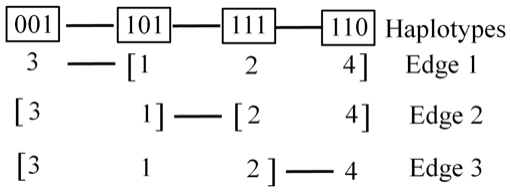
Illustration of tree topology example described in text.

Parsimony trees for the 4gamete haplotypes were estimated using the pars function in Phylip [40]. Blocks produced by the HapBlock and SlideWin3 methods were unsuitable because they could include significant amounts of recombination that would result in failure to find a single best tree. The parsimony tree file for each 4gamete block was analyzed by the program TreeScan [27], which identifies the edges in the tree and, for each edge, the alleles that belong to the two clades defined by the edge. For example, if a block has four alleles (101,111,001,110), the tree topology is (3∶1.00,(4∶1.00,2∶0.00)∶1.00,1∶0.00) and there are three edges in the tree: 3 / 4 2 1; 4 2 / 3 1; 4 / 3 2 1. This is illustrated in Figure 4.

Using the TreeScan output, a clade membership probability file was created as follows: 2 columns were created for each of the *k* edges at each 4gamete block. The first column for each edge stores the probability that the individual belongs to the first clade at that edge. The second column stores the probability that the individual belongs to neither clade; this occurs when an individual carries a rare haplotype that wasn't included in the tree. As for the other haplotype methods, unblocked SNPs were represented by one column each.

### Phenotype simulation

Simulated phenotypes were composed of one QTL, a polygenic effect, and error. Different QTL sizes (*p* = 0.03, 0.06, and 0.12 of phenotypic variation) and heritability (*h^2^* = 0.25 and 0.75) were simulated. One hundred SNPs, chosen to minimize pairwise LD and to maximize genetic map coverage, were designated as quantitative trait nucleotides (QTLs) and removed from the marker dataset, leaving 2098 markers for the association mapping analysis. Minor allele frequency among the 100 QTL was at least 0.10; median MAF was 0.34. Simulated QTL were assigned an additive effect inversely proportional to the standard deviation of the allelic state in order to standardize the amount of genetic variance attributed to QTL with different MAF. SNPs composing the polygenic effect were selected by forming 400 *k*-means [41] clusters of SNP markers from Barley CAP genotype data and selecting the SNP nearest the centroid of each cluster. This step ensured that more than one SNP per haplotype block was not used for the polygenic effect. Marker scores at these loci were simply summed for each line to create the polygenic component of the phenotype.

Variation due to the QTL and polygenic component together compose the total genetic variance. Error effects were randomly sampled from a standard normal distribution and added to the genetic values to obtain phenotypes with *h^2^* = 0.75 or 0.25. Each of the 100 SNPs designated as QTL was used to simulate 10 phenotypes, producing 1000 phenotypes. To summarize, a factorial of QTL size (*p* = 3, 6, and 12% of phenotypic variation) and *h^2^* (0.75 and 0.25) was used to form six sets of 1000 phenotypes.

Because we were concerned that simulating QTL using SNPs from the Barley CAP genotyping platform favored the single SNP analysis, we also simulated QTL alleles that deviated from the single SNP model in terms of frequency and LD with surrounding SNPs. This was accomplished by defining a QTL effect as a particular combination of adjacent SNPs. The pairs of adjacent SNPs (hereafter referred to as QTL pair) were chosen on the basis of each haplotype block structure so that each haplotype block method would be favored by one set of simulated QTL. Designating QTL pairs based on the SlideWin3 block structure, an effect was given to individuals with the allelic combination (0-0) at the first two positions within each 3-SNP haplotype block. In Figure 4, this would correspond to Edge 1, which separates alleles 001 from all other alleles. To simulate QTL based on the 4gamete block structure, an effect was given to individuals with a specific pair of alleles at the center of the blocks as defined by the 4gamete method. Because there are only 791 4gamete haplotype blocks and therefore only 791 QTL pairs, another allelic combination (0-1) was used to designate additional QTL pair effects. QTL pair designation on the basis of HapBlock haplotype blocks was carried out like that for 4gamete. In all three cases, allele combinations with frequency less than 2.8% were not considered. As before, single SNPs were used to simulate QTL, but in this case the causal SNPs were left in the marker dataset so a marker in complete LD with the QTL always existed. The remainder of the phenotype was simulated as described above.

### Selection of lines included in simulation study

A population of 400 lines was selected from the 1807 original lines by forming 400 *k*-means clusters based on the SNP data and selecting the line nearest the centroid. If two or more lines were equidistant from the centroid, one of these lines was randomly selected. Population size was reduced from 1807 to 400 to reduce computing time. Additionally, a population size of 400 is within the range of population sizes typically used in association genetics studies [42]. Creating 400 *k*-means clusters and sampling the line nearest the centroid maximized allelic diversity and historical recombination within the selected lines. Maximizing genetic diversity is often a primary objective in designing association mapping panels [43], [44].

### Association mapping analysis

For QTL detection, marker-trait associations were tested by the mixed linear model
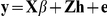
where **y** is a vector of phenotypes, 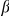 is a vector of fixed marker effects (i.e., single SNP, haplotype alleles, or parsimony tree edges), **h** is a vector of polygenic effects caused by relatedness, **e** is vector of residual effects, and **X** and **Z** are incidence matrices relating **y** to 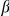 and **y** to **h**, respectively. It is assumed **h**∼N(0, 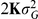) and **e**∼N(0, 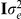), where **K** is an allele-sharing matrix calculated from the SNP data, 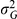 is the genetic variance, **I** is an identity matrix, and 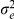 is the residual variance. Zhao et al. [2] found that modeling population structure with an allele-sharing matrix controlled false positives as well as using a mixed model including both a kinship matrix and population substructure effect (Q matrix) used by Yu et al. [45].

The above model was implemented using the efficient mixed-model association algorithm (EMMA) of Kang et al. [46]. The most important advantage of EMMA for our purposes is its speed, being orders of magnitude faster than other mixed model algorithms. An allele-sharing matrix was calculated using the *kinship.emma* function. As it is currently set up, the EMMA R package has strict formatting requirements and handles only bi-allelic marker data. In order to use EMMA to analyze the 4gamete, HapBlock, and SlideWin3 data, we modified the EMMA R package to perform likelihood-ratio tests of more than one degree of freedom (df). The haplotype allele incidence matrix, as described in *Haplotype Block Identification* section, is used in place of the SNP matrix. A likelihood-ratio test was performed for each haplotype block, and the test statistic was compared to a chi-squared distribution with df equal to the number of haplotypes at the locus in question minus one. To validate our modified EMMA R script, one phenotype was analyzed using the above model and 4gamete haplotype data in PROC MIXED of SAS by performing a likelihood-ratio test for each locus. The resulting p-value of each 4gamete haplotype block was the same across all markers as those obtained from the modified EMMA script.

The format of the TreeScan edge score data is exactly that of single SNP data, allowing use of the unmodified EMMA package. While the format is the same, it is important to note that each TreeScan test is for the effect of a tree edge on the phenotype. That is, lines are grouped by their clade membership within the parsimony tree created for each 4gamete block and clades connected by an edge are tested for phenotype differences. This obviously can result in several tests per haplotype block. The between-clade test resulting in the lowest p-value is retained as the representative test of any given haplotype block. This results in matrix of p-values with the same dimensions as the 4gamete p-value matrix.

### Power and false discovery rate

Comparing performance between the five different marker types – single SNP, 4gamete, HapBlock, SlideWin3, TreeScan edges – was done on the basis of power at empirical FDR of 0.10 and 0.20. A 20 cM window surrounding the QTL was used for declaring a true discovery. For the haplotype markers, distance to the QTL was calculated from the borders of the block. When the QTL was simulated as a combination of two QTLs, the QTL position was set to the midposition of the QTL pair. At any given p-value, power is calculated as the number of times at least one association is found within 10 cM of the true QTL position divided by 1000 (because there were 1000 single-QTL simulations). False discovery rate was calculated as 1−(*true associations*)/(*total associations*), where *true associations* is the total number of marker-phenotype associations declared significant at a nominal p-value where the marker in question was located within 10 cM of the QTL and *total associations* is the total number of marker-phenotype associations declared significant regardless of position relative to the QTL. A superior marker type for association mapping maximizes the power at the given FDR.

To estimate power and its standard error at an empirical FDR of 0.10 or 0.20, the following bootstrapping algorithm was performed: 1) p-values from the association analysis were arranged in a 1000 by *m* matrix, where 1000 is equal to the number of phenotypes analyzed and *m* is equal to the number of markers of the method used (e.g., *m* = 2098 for single SNPs and *m* = 791 for 4gamete; Table 2). 2) 1000 rows (i.e., simulated phenotypes) of this matrix were randomly sampled with replacement. 3) Power and FDR were calculated as described above. 4) Power at a specific FDR was interpolated using a local simple linear regression model relating power to FDR. 5) Steps 2–4 were repeated 1000 times to produce a bootstrap sampling distribution of power at a designated FDR. 6) A point estimate of power was taken as the mean of the bootstrap sample distribution and the standard error was calculated as the standard deviation of the bootstrap sampling distribution. Bootstrap sample power estimates were normally distributed in all cases.

### Heading date analysis

Each year of the Barley CAP, every participating breeding program contributed 96 lines for genotyping and phenotyping. Phenotypic evaluations were conducted at one or more locations within the breeding program's geographical region. Evaluations were conducted during one year only, i.e. lines contributed in a specific year are evaluated during that year's field season weather permitting. Hence, the phenotypic data of the Barley CAP is highly unbalanced.

Heading date (days after planting when 50% of panicles emerged from flag leaf) data sets of spring barley were obtained from The Hordeum Toolbox (THT; www.hordeumtoolbox.org). If a set of CAP lines (96) was evaluated at more than one location, data was combined across locations and repeatability on an entry-mean basis was calculated as 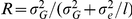 where *l* is the number of locations. Data sets from individual breeding programs were included if 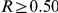 to ensure high data quality. If a breeding program only used one location, data from that program was included if the reported coefficient of variation on THT was less than 10%. After selecting datasets according to the above criteria, the remaining heading date data was from five programs: Aberdeen, ID; University of Minnesota; North Dakota State University (NDSU) 6-row; NDSU 2-row; Washington State University. Data from both 2006 and 2007 (i.e., 192 lines from each program) was used from each program.

Best linear unbiased predictions (BLUPs) of line performance were calculated using mixed model involving trial as a fixed effect and line as a random effect. The trial variable corresponds to the individuals locations used by different breeding programs and was modeled because trials within breeding programs were often unbalanced. Check varieties common between locations within breeding programs and across breeding programs provided the overlap needed so that line BLUPs could be adjusted for environmental effects. Outliers were identified as those with a standardized value greater than three standard deviations and removed from the dataset. The number of lines remaining was 944.

The statistical model used for association mapping of heading date was the same as that used for simulated phenotypes except for the inclusion of breeding program as a fixed effect. This model effectively associated within-breeding program genetic variation with markers, removing any unbalanced environmental effects that could cause false phenotype-marker associations. A statistical threshold corresponding to an experiment-wise type I error rate of 0.05 was established for each marker method by randomly permuting the phenotypes 1000 times.

## References

[1] [Anon], Wellcome Trust Case Control Consortium (2007). Genome-wide association study of 14,000 cases of seven common diseases and 3,000 shared controls.. Nature.

[2] Zhao KY, Aranzana MJ, Kim S, Lister C, Shindo C (2007). An arabidopsis example of association mapping in structured samples.. Plos Genetics.

[3] Parker HG, Vonholdt BM, Quignon P, Margulies EH, Shao S (2009). An expressed Fgf4 retrogene is associated with breed-defining chondrodysplasia in domestic dogs.. Science (Wash ).

[4] Charlier C, Coppieters W, Rollin F, Desmecht D, Agerholm JS (2008). Highly effective SNP-based association mapping and management of recessive defects in livestock.. Nat Genet.

[5] Gabriel SB, Schaffner SF, Nguyen H, Moore JM, Roy J (2002). The structure of haplotype blocks in the human genome.. Science.

[6] Zhao H, Pfeiffer R, Gail MH (2003). Haplotype analysis in population genetics and association studies.. Pharmacogenomics.

[7] Clark AG (2004). The role of haplotypes in candidate gene studies.. Genet Epidemiol.

[8] Bardel C, Danjean V, Hugot JP, Darlu P, Genin E (2005). On the use of haplotype phylogeny to detect disease susceptibility loci.. Bmc Genetics.

[9] Meuwissen TH, Goddard ME (2000). Fine mapping of quantitative trait loci using linkage disequilibria with closely linked marker loci.. Genetics.

[10] Templeton AR, Boerwinkle E, Sing CF (1987). A cladistic-analysis of phenotypic associations with haplotypes inferred from restriction endonuclease mapping .1. basic theory and an analysis of alcohol-dehydrogenase activity in drosophila.. Genetics.

[11] Morris RW, Kaplan NL (2002). On the advantage of haplotype analysis in the presence of multiple disease susceptibility alleles.. Genet Epidemiol.

[12] Dimas AS, Stranger BE, Beazley C, Finn RD, Ingle CE (2008). Modifier effects between regulatory and protein-coding variation.. Plos Genetics.

[13] Hamon SC, Kardia SL, Boerwinkle E, Liu K, Klos KL (2006). Evidence for consistent intragenic and intergenic interactions between SNP effects in the APOA1/C3/A4/A5 gene cluster.. Hum Hered.

[14] Long AD, Langley CH (1999). The power of association studies to detect the contribution of candidate genetic loci to variation in complex traits.. Genome Res.

[15] Hayes BJ, Chamberlain AJ, McPartlan H, Macleod I, Sethuraman L (2007). Accuracy of marker-assisted selection with single markers and marker haplotypes in cattle.. Genet Res.

[16] Calus MP, Meuwissen TH, Windig JJ, Knol EF, Schrooten C (2009). Effects of the number of markers per haplotype and clustering of haplotypes on the accuracy of QTL mapping and prediction of genomic breeding values.. Genet Sel Evol.

[17] Grapes L, Dekkers JCM, Rothschild MF, Fernando RL (2004). Comparing linkage disequilibrium-based methods for fine mapping quantitative trait loci.. Genetics.

[18] Zhao HH, Fernando RL, Dekkers JCM (2007). Power and precision of alternate methods for linkage disequilibrium mapping of quantitative trait loci.. Genetics.

[19] Shim H, Chun H, Engelman CD, Payseur BA (2009). Genome-wide association studies using single-nucleotide polymorphisms versus haplotypes: An empirical comparison with data from the north american rheumatoid arthritis consortium.. BMC Proc.

[20] Lu X, Niu TH, Liu JS (2003). Haplotype information and linkage disequilibrium mapping for single nucleotide polymorphisms.. Genome Res.

[21] Hagenblad J, Tang CL, Molitor J, Werner J, Zhao K (2004). Haplotype structure and phenotypic associations in the chromosomal regions surrounding two arabidopsis thaliana flowering time loci.. Genetics.

[22] Reich DE, Cargill M, Bolk S, Ireland J (2001). Linkage disequilibrium in the human genome.. Nature.

[23] Khatkar MS, Nicholas FW, Collins AR, Zenger KR (2008). Extent of genome-wide linkage disequilibrium in Australian Holstein-Friesian cattle based on a high-density SNP panel.. BMC Genomics.

[24] Hamblin MT, Close TJ, Bhat PR (2010). Population structure and linkage disequilibrium in US barley germplasm: Implications for association mapping.. Crop Science.

[25] Barrett JC, Fry B, Maller J, Daly MJ (2005). Haploview: Analysis and visualization of LD and haplotype maps.. Bioinformatics.

[26] Zhang K, Deng MH, Chen T, Waterman MS, Sun FZ (2002). A dynamic programming algorithm for haplotype block partitioning.. Proc Natl Acad Sci U S A.

[27] Posada D, Maxwell TJ, Templeton AR (2005). TreeScan: A bioinformatic application to search for genotype/phenotype associations using haplotype trees.. Bioinformatics.

[28] Close TJ, Bhat PR, Lonardi S, Wu YH, Rostoks N (2009). Development and implementation of high-throughput SNP genotyping in barley.. BMC Genomics.

[29] Horsley RD, Schmierer D, Maier C, Kudrna D, Urrea CA (2006). Identification of QTLs associated with fusarium head blight resistance in barley accession CIho 4196.. Crop Sci.

[30] Nduulu LM, Mesfin A, Muehlbauer GJ, Smith KP (2007). Analysis of the chromosome 2(2H) region of barley associated with the correlated traits fusarium head blight resistance and heading date.. Theor Appl Genet.

[31] Hayes PM, Liu BH, Knapp SJ, Chen F, Jones B (1993). Quantitative trait locus effects and environmental interaction in a sample of north-american barley germ plasm.. Theor Appl Genet.

[32] Thomas WTB, Powell W, Waugh R, Chalmers KJ, Barua UM (1995). Detection of quantitative trait loci for agronomic, yield, grain and disease characters in spring barley (hordeum-vulgare L).. Theor Appl Genet.

[33] Pillen K, Zacharias A, Leon J (2004). Comparative AB-QTL analysis in barley using a single exotic donor of hordeum vulgare ssp spontaneum.. Theor Appl Genet.

[34] Yan L, Fu D, Li C, Blechl A, Tranquilli G (2006). The wheat and barley vernalization gene VRN3 is an orthologue of FT.. Proc Natl Acad Sci U S A.

[35] Zhao H, Nettleton D, Soller M, Dekkers JC (2005). Evaluation of linkage disequilibrium measures between multi-allelic markers as predictors of linkage disequilibrium between markers and QTL.. Genet Res.

[36] Schaid DJ (2004). Evaluating associations of haplotypes with traits.. Genet Epidemiol.

[37] Purcell S, Neale B, Todd-Brown K, Thomas L, Ferreira MAR (2007). PLINK: A tool set for whole-genome association and population-based linkage analyses.. Am J Hum Genet.

[38] Bradbury PJ, Zhang Z, Kroon DE, Casstevens TM, Ramdoss Y (2007). TASSEL: Software for association mapping of complex traits in diverse samples.. Bioinformatics.

[39] Scheet P, Stephens M (2006). A fast and flexible statistical model for large-scale population genotype data: Applications to inferring missing genotypes and haplotypic phase.. Am J Hum Genet.

[40] Felsenstein J (2009). PHYLIP (phylogeny inference package)..

[41] Hartigan JA (1975). Clustering algorithms.

[42] Zhu CS, Gore M, Buckler ES, Yu JM (2008). Status and prospects of association mapping in plants.. Plant Genome.

[43] Flint-Garcia SA, Thuillet AC, Yu JM, Pressoir G, Romero SM (2005). Maize association population: A high-resolution platform for quantitative trait locus dissection.. Plant Journal.

[44] Casa AM, Pressoir G, Brown PJ, Mitchell SE, Rooney WL (2008). Community resources and strategies for association mapping in sorghum.. Crop Sci.

[45] Yu JM, Pressoir G, Briggs WH, Bi IV, Yamasaki M (2006). A unified mixed-model method for association mapping that accounts for multiple levels of relatedness.. Nat Genet.

[46] Kang HM, Zaitlen NA, Wade CM, Kirby A, Heckerman D (2008). Efficient control of population structure in model organism association mapping.. Genetics.

